# Supplementary material for: Dissociable components of the reward circuit are involved in appraisal versus choice
Source: Sci Rep. 2019 Feb 13;9:1958. doi: 10.1038/s41598-019-38927-7 (PMC6374444; doi:10.1038/s41598-019-38927-7)
Supplement: Supplementary file 1 — Supplementary Information (final) [file 41598_2019_38927_MOESM1_ESM.pdf]

Dissociable components of the reward circuit are involved in appraisal versus choice

Amitai Shenhav & Uma R. Karmarkar

Supplementary Information

Supplementary Methods

1. Confirmatory whole-brain analyses.

We supplemented our ROI analyses with whole-brain GLMs. For these analyses, first-level GLMs again separately modeled events occurring at Evaluation and Selection periods of each trial. The Evaluation period was modeled as a single event, modulated by a parameter of interest, including (log) evaluation time, task condition (indicator variable for Choose vs. Like), set liking, and decision certainty. Parametric regressors were not orthogonalized with respect to one another, allowing them to compete for variance independently. Missed trials (failures to choose a response within 5s in the Selection period) occurred rarely (0.4% of trials) and were modeled as a separate condition. As with our primary analyses, trials were concatenated and appropriate regressors were included to account for block-wise effects, and the GLMs were estimated with RobustWLS. We performed second-level random-effects analyses on the beta estimates generated at the first level, and whole-brain group statistical maps were generated using one-sample t-tests over these contrasts. These maps were extent-thresholded to achieve a whole-brain family-wise error cluster-corrected $p<0.05$. To avoid obscuring areas of potential overlap, we used a cluster-forming threshold of $p<0.005$ to generate these maps. However, despite the fact that these maps are confirmatory and therefore not used as the basis for statistical inference, in order to guard against potential false-positives[1,2] we separately confirmed that the same clusters remain significant with a more conservative cluster-forming threshold of $p<0.001$. These maps were projected onto the Caret-inflated cortical surface[3].

Supplementary Results

1. Alternate value difference formulation

Our analyses estimate decision certainty on Choose trials based on the absolute difference between the value of the chosen item and the average of the remaining items. We considered whether a better estimate might obtain from the difference between the chosen item and the next-best item. While this alternate, Chosen-Versus-Next variable also correlates negatively with RT ($\beta = -0.19$, $t(26.0) = -7.3$, $p < 0.001$) and positively with choice confidence ($\beta = 0.14$, $t(26.0) = -6.9$, $p < 0.001$), these effects were overall smaller than those observed for the Chosen-Versus-Remaining variable. When entering both into the same regression, we find that only Chosen-Versus-Remaining continues to predict RT ($\beta = -0.27$, $t(37.2) = -8.1$, $p < 0.001$) and confidence ($\beta = 0.25$, $t(27.8) = 6.2$, $p < 0.001$) (Chosen-Versus-Next $|ts| < 2.0$, $p > 0.05$).

2. Tests for other decision value signals in Dorsal and Ventral Value Networks

To confirm the specificity of our results relating the Dorsal and Ventral Value Networks to Like and Choose respectively, we ran separate regressions to test whether either network demonstrated other value-related signals during either task or across tasks. Focusing on the appraisal task or collapsing across all trials, we did not find significant correlates of the overall set value, the value of the set's chosen option, or the difference between the chosen and unchosen options (Table S2). Focusing these analyses on Choose trials, we found positive correlates of chosen value and value difference in the Dorsal Value Network, but not the Ventral Value Network (Table S2). However, because chosen value and value difference are correlated ($r = 0.58$), we included them together in a single GLM and found that activity in the dorsal network on Choose trials is only significantly associated with value difference (consistent with the decision certainty analyses above; $\beta = 0.10$, $p < 0.05$) and not otherwise associated with the value of the chosen item ($p = 0.64$) or the overall set value ($p = 0.39$) (Table S4). None of these variables were significant predictors of activity in the Ventral Value Network (Table S4).

3. Analysis of subjective choice confidence

We also tested whether either network tracked subjective ratings of confidence for Choose decisions taken at the end of the experiment (during either task and across tasks), and did not find any such correlates ($|t|<1.1$, $p>0.30$). However, in addition to being retrospective, we note that these ratings were heavily skewed towards ceiling-level confidence ratings (mean = 4.25 [out of 5], SD = 1.06, median = 5). This was one of the motivations for focusing our decision certainty analyses on a continuous estimate of Choose certainty (value difference) that had a direct analog on Like trials (rating extremity), both of which having been measured at decision time.

Supplementary Figures

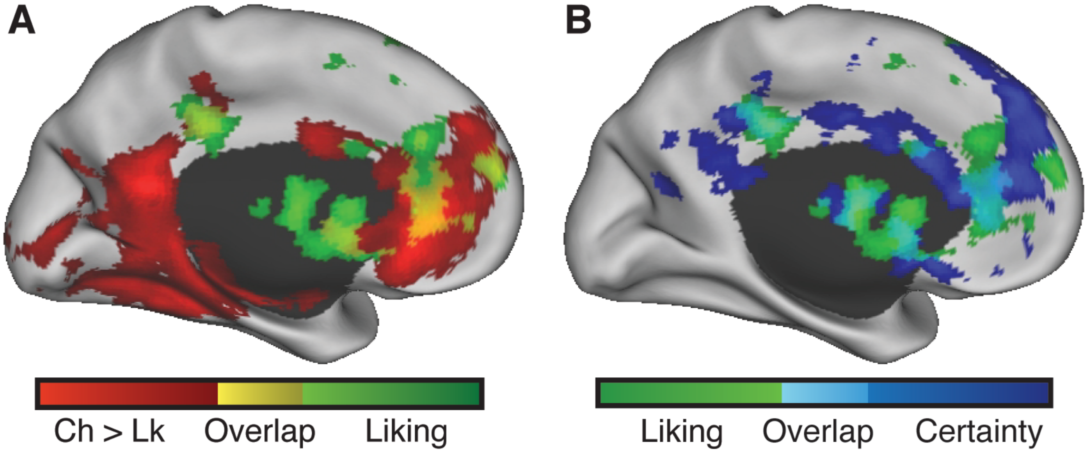

Figure S1. **A)** Confirmatory whole-brain analyses show that ventral striatum and more dorsal regions of vmPFC and PCC tracked set liking across tasks (green), whereas more ventral regions of vmPFC/PCC was significantly more active for Choose relative to Like trials (red), overlapping the set liking network (yellow). **B)** Decision certainty also activated regions of ventral striatum and dorsal regions of vmPFC/PCC (blue), overlapping regions associated with set liking (turquoise). Whole-brain statistical maps are thresholded at a cluster-corrected family-wise error (FWE) p<0.05.

Supplementary Tables

Choose > Like					
Region	Cluster-level p-value	Cluster size (voxels)	Peak Voxel Z-score	Peak Voxel p-value	MNI Coordinates (mm)
L Retrosplenial, parahippocampal cortex, calcarine, LOC	<0.00001	10051	5.98	<0.00001	-6,-56,18
vmPFC, L MFG	<0.00001	4704	5.70	<0.00001	-6,38,0
L Lateral OFC	0.043	283	5.08	<0.00001	-34,32,-14
R STG, operculum	0.0018	498	4.87	<0.00001	48,-8,-8
R Calcarine, LOC	<0.00001	1308	4.81	<0.00001	18,-78,12
L Posterior insula	0.036	294	4.29	<0.00001	-40,-4,-8
B Cerebellum	0.039	290	3.75	0.00009	2,-54,-36

Like > Choose					
Region	Cluster-level p-value	Cluster size (voxels)	Peak Voxel Z-score	Peak Voxel p-value	MNI Coordinates (mm)
R DLPFC, IFG	<0.00001	3664	5.43	<0.00001	46,38,22
R IPL	<0.00001	1039	5.25	<0.00001	50,-36,52
L IFG	0.0026	470	4.81	<0.00001	-48,14,6
L IPL	<0.00001	954	4.69	<0.00001	-36,-44,44
PCC	0.023	322	4.38	<0.00001	-4,-24,28
R MTG	0.044	282	4.35	<0.00001	58,-46,-8
R SMA, SFG	0.0053	421	3.75	0.00009	6,28,48

Set liking (all trials)					
Region	Cluster-level p-value	Cluster size (voxels)	Peak Voxel Z-score	Peak Voxel p-value	MNI Coordinates (mm)
B VS/caudate, pgACC, L DLPFC, L MFG	<0.00001	7532	5.10	<0.00001	-34,6,58
L MTG	0.0000614	806	5.02	<0.00001	-58,-44,-16
B PCC	0.0164	362	4.40	<0.00001	6,-32,36
L IPL	0.00012	750	4.05	0.00003	-44,-46,44

Certainty (all trials)					

Region	Cluster-level p-value	Cluster size (voxels)	Peak Voxel Z-score	Peak Voxel p-value	MNI Coordinates (mm)
B VS/caudate, pgACC, PCC, R IFG	<0.00001	10728	4.96	<0.00001	-4,46,18
L MTG	<0.00001	2526	4.74	<0.00001	-56,-24,-12
R amygdala	0.0022	477	4.55	<0.00001	26,-2,-18
L precentral, IFG	<0.00001	1252	4.05	0.00003	-10,-8,58
R precentral	0.033	297	3.85	0.00006	44,-8,58
L postcentral	0.016	343	3.64	0.00014	-44,-28,44
R MTG	0.00013	691	3.62	0.00015	52,-54,14
R TPJ	<0.00001	1026	3.58	0.00017	50,-8,22

Table S1. Results of whole-brain analysis for task (Choose vs. Like), set liking, and decision certainty. All of these were included in the same GLM, which also covaried RT. Whole-brain maps were thresholded at a voxelwise $p<0.005$, extent-thresholded to obtain a clusterwise $p<0.05$. LOC: lateral occipital cortex, MFG: middle frontal gyrus, STG: superior temporal gyrus, DLPFC: dorsolateral prefrontal cortex, IFG: inferior frontal gyrus, SFG: superior frontal gyrus, IPL: inferior parietal lobule, MTG: middle temporal gyrus, SMA: supplementary motor area, TPJ: temporoparietal junction.

Network	Task	Predictor	β	SEM	t	p
Dorsal Value Network	All trials	Overall value	0.04	0.02	1.68	0.106
		Chosen value	0.04	0.02	1.91	0.067
		Value difference	0.03	0.02	1.59	0.12
		Signed VD	0.02	0.02	1.17	0.24
	Like trials	Overall value	0.03	0.03	0.98	0.335
		Chosen value	0.00	0.03	0.10	0.919
		Value difference	-0.04	0.02	-1.75	0.08
		Signed VD	-0.04	0.03	-1.41	0.16
	Choose trials	Overall value	0.05	0.03	1.75	0.092
		Chosen value	0.08	0.03	3.06	0.005
		Value difference	0.10	0.03	3.84	0.0008
		Signed VD	0.07	0.02	2.92	0.003
Ventral Value Network	All trials	Overall value	0.03	0.02	1.59	0.124
		Chosen value	0.02	0.02	0.82	0.421
		Value difference	0.00	0.02	-0.23	0.817
		Signed VD	-0.01	0.02	-0.84	0.403
	Like trials	Overall value	0.02	0.03	0.63	0.536
		Chosen value	0.00	0.03	0.08	0.939
		Value difference	-0.02	0.02	-0.93	0.354
		Signed VD	-0.02	0.02	-0.71	0.48
	Choose trials	Overall value	0.05	0.03	1.76	0.087
		Chosen value	0.03	0.03	1.12	0.270
		Value difference	0.01	0.03	0.34	0.738
		Signed VD	-0.01	0.03	-0.53	0.60

Table S2. Regression estimates for regressions predicting BOLD activity in the Ventral and Dorsal Value Networks based on overall value (average item value), chosen value (value of the item chosen from the set during the Choose task), (unsigned) value difference (VD; absolute distance between the chosen value and the average value of the remaining items), and signed VD (chosen value minus average value of the remaining items). Each row represents a separate regression involving a single predictor. See the main text for relevant simultaneous regressions.

**Outcome**	**Predictor**	**β**	**SEM**	**t**	**p**
**Set liking**					
	pgACC	0.05	0.02	2.42	0.017
	VS	0.07	0.02	3.29	<0.005
	PCC	-0.02	0.02	-0.72	0.47
	Evaluation time	-0.05	0.02	-2.03	0.053
**Task condition**					
	mOFC	0.16	0.06	2.72	<0.01
	MFG	0.16	0.07	2.43	0.015
	RSC	0.17	0.05	3.09	<0.005
	Evaluation time	0.24	0.07	3.32	<0.001

Table S3. Regression estimates for regressions predicting set liking and task condition based on individual sub-regions within the Dorsal Value Network (pgACC, VS, PCC) and Ventral Value Network (mOFC, MFG, RSC), respectively, covarying evaluation time.

**Outcome**	**Predictor**	**β**	**SEM**	**t**	**p**
**Dorsal Value Network** (Choose Trials)					
	Overall value	0.04	0.05	0.86	0.39
	Chosen value	0.01	0.02	0.47	0.64
	Value difference	0.10	0.04	2.47	0.02
	Evaluation time	0.04	0.03	1.20	0.24
**Ventral Value Network** (Choose Trials)					
	Overall value	0.06	0.05	1.25	0.21
	Chosen value	-0.00	0.02	-0.06	0.95
	Value difference	0.04	0.04	0.87	0.39
	Evaluation time	0.09	0.03	3.12	<0.005

Table S4. Regression estimates for Dorsal Value Network and Ventral Value Network activity on Choose trials, based on overall value, chosen value, value difference, and evaluation time.

**Outcome**	**Predictor**	**β**	**SEM**	**t**	**p**
**Dorsal Value Network**					
	Condition	0.10	0.04	2.57	0.011
	Set liking	0.08	0.03	2.93	0.007
	Decision certainty	0.05	0.02	2.35	0.020
	Condition*liking	-0.03	0.04	-0.74	0.46
	Condition*certainty	0.04	0.05	0.91	0.37
	Evaluation time	0.05	0.02	2.73	0.009
**Ventral Value Network**					
	Condition	0.15	0.04	3.86	<0.001
	Set liking	0.07	0.03	2.51	0.016
	Decision certainty	0.01	0.02	0.60	0.55
	Condition*liking	-0.01	0.04	-0.24	0.81
	Condition*certainty	0.06	0.05	1.20	0.24
	Evaluation time	0.07	0.02	3.67	<0.001

Table S5. Regression estimates for regressions predicting BOLD activity at the onset of the options (prior to task cue onset) in the Dorsal and Ventral Value Networks based on simultaneous predictors for task condition (Choose > Like), set liking, (task-relevant) decision certainty, condition x liking interaction, condition x certainty interaction, and (log) evaluation time.

Supplementary References

1 Eklund, A., Nichols, T. E. & Knutsson, H. Cluster failure: why fMRI inferences for spatial extent have inflated false-positive rates. *Proc. Natl. Acad. Sci.* **113**, 7900-7905, (2016).

2 Woo, C. W., Krishnan, A. & Wager, T. D. Cluster-extent based thresholding in fMRI analyses: pitfalls and recommendations *NeuroImage* **91**, 412-419, (2014).

3 Van Essen, D. A population-average, landmark-and surface-based (PALS) atlas of human cerebral cortex. *NeuroImage* **28**, 635-662, (2005).
